# Increased comparability between RNA-Seq and microarray data by utilization of gene sets

**DOI:** 10.1371/journal.pcbi.1008295

**Published:** 2020-09-30

**Authors:** Frans M. van der Kloet, Jeroen Buurmans, Martijs J. Jonker, Age K. Smilde, Johan A. Westerhuis

**Affiliations:** Swammerdam Institute for Life Sciences, University of Amsterdam; University of Virginia, UNITED STATES

## Abstract

The field of transcriptomics uses and measures mRNA as a proxy of gene expression. There are currently two major platforms in use for quantifying mRNA, microarray and RNA-Seq. Many comparative studies have shown that their results are not always consistent. In this study we aim to find a robust method to increase comparability of both platforms enabling data analysis of merged data from both platforms. We transformed high dimensional transcriptomics data from two different platforms into a lower dimensional, and biologically relevant dataset by calculating enrichment scores based on gene set collections for all samples. We compared the similarity between data from both platforms based on the raw data and on the enrichment scores. We show that the performed data transforms the data in a biologically relevant way and filters out noise which leads to increased platform concordance. We validate the procedure using predictive models built with microarray based enrichment scores to predict subtypes of breast cancer using enrichment scores based on sequenced data. Although microarray and RNA-Seq expression levels might appear different, transforming them into biologically relevant gene set enrichment scores significantly increases their correlation, which is a step forward in data integration of the two platforms. The gene set collections were shown to contain biologically relevant gene sets. More in-depth investigation on the effect of the composition, size, and number of gene sets that are used for the transformation is suggested for future research.

## Author summary

The field of transcriptomics uses and measures mRNA as a proxy of gene expression. There are currently two major platforms in use for quantifying mRNA, microarray and RNA-Seq. Many comparative studies have shown that their results are not always consistent. In this study we aim to find a robust method to increase comparability of both platforms enabling data analysis of merged data from both platforms. We transformed the high dimensional transcriptomics data from the two different platforms into lower dimensional, and biologically relevant gene set scores. These gene sets were defined a-priori as specific combination of genes (e.g. up-regulated in a certain pathway). We observed that although microarray and RNA-Seq expression levels might appear different, using these gene sets to transform the data significantly increases their correlation. This is a step forward in data integration of the two platforms. More in-depth investigation on the effect of the composition, size, and number of gene sets that are used for the transformation is suggested for future research.

## Introduction

To determine cellular activity of a culture or tissue, the field of transcriptomics currently has two major platforms at its disposal, namely microarrays and RNA-Seq. As a proxy of gene expression both platforms can be used to quantify the constituent of all protein encoding transcripts, or mRNA, present in a sample. Because mRNA has a high rate of decay, its momentary composition can be considered a snapshot of gene activity. Analysis of the transcriptome of a cell in different conditions or time points can therefore give invaluable insights in the differences on a molecular level between healthy or diseased tissues or the response to external stimuli like drugs or stress.

A microarray consists of probes that are composed of many specific single strand DNA fragments, which makes the probe sensitive to the complementary sequence. The microarray method relies on reverse transcriptases to convert mRNA, isolated from the sample, back into DNA (cDNA) while introducing fluorescent labeled nucleotides [1]. Quantification of the different transcripts is performed by allowing the fluorescently labeled fragment library to hybridize to the probes on the microarray slide. The level of fluorescence is measured per probe which is thereby a relative measure for the number of probe specific transcripts that were present in the sample. Microarrays have been in use as a transcriptomics platform since 1995 while the newer high throughput RNA-Seq method was only used as such since 2008 [2]. RNA-Seq is a next generation sequencing (NGS) method that, other than its name suggests, sequences DNA. To determine mRNA levels, a cDNA fragment library preparation step is needed, analogous to microarray analysis [3]. The clonal cDNA fragments are individually sequenced to the single stranded cDNA. This fragment is then sequenced by a process called sequencing by synthesis (lllumina). In this process the complementary strand is extended with a single nucleotide per cycle. The type of nucleotide (A, C, T, G) that was incorporated in the strand is determined by a fluorescent label which is then cleaved off, this in turn allows extension by a subsequent nucleotide. This cycle is repeated in a massively parallel fashion. The sequenced fragments are then mapped to a reference genome. If such a reference genome is not available, *de novo* transcriptome assembly is possible depending on adequate coverage and sequence depth.

RNA-Seq is associated with a higher dynamic range than microarrays [4, 5] making it suitable for detection of low abundant transcripts. Furthermore, as RNA-Seq is not necessarily reliant on a reference genome this platform allows for novel transcript detection and transcriptomics analysis for organisms for which no reference genome is available. These advantages have made RNA-Seq gain in popularity leading to a reduction of the overall cost of the platform which is expected to fully replace the microarray platform. Although most future gene expression studies will use an RNA-Seq platform, it would be a waste of effort and resources if all the microarray datasets available would be ignored from now on. Meta-analysis of gene expression over multiple studies independent on the type of platform would increase sample size and power of such analyses. This however, requires a high comparability between data from the two platforms.

The question about comparability between the microarray platform and RNA-Seq platform has received attention in previous papers. Fu et al. [6] compared microarray and RNA-Seq platforms and found correlation values of 0.62 up to 0.75 for microarray and RNA-Seq measurements of groups of around 8,000 and 5000 selected genes. Zhao et al. [5] focused on the differences between the two platforms and showed how background hybridisation and probe saturation in microarrays resulted in limited sensitivity in both low and high expression levels. Meta-analysis ([7, 8]) for combined microarray and RNA-Seq studies therefore seems to be more problematic. Jung et al. used rank products to combine RNA-seq and microarray data for exploring carcinogenic risk [9]. Training Distribution matching (TDM) was introduced by Thompson et al. [7] in which one of the datatypes (RNA-seq or microarray) is transformed in such a way that it can be used with a model developed on the other data type.

Hänzelmann et al [8] compare their method to other implementations of enrichment score based methods (PLAGE, ssGSEA and combined z-score) but using either only micro-array or sequencing data to discover subtle pathway activity changes. In this paper we explore single sample GSEA (ssGSEA) related to GSEA [10], to make gene expression data from microarray and RNA-Seq more comparable on an individual sample level. We combine single gene expression values for a specific set of genes (*a priori* defined) into a single enrichment score for that set. This way multiple gene sets together (called gene set collections), are used to transform the genes into a smaller collection of gene set enrichment scores for every sample. The enrichment score represents the degree to which the genes within each set are expressed, i.e. all over or under expressed. We propose that these enrichment scores can be treated as a new “latent variable” and that the conversion to enrichment scores can be considered as a form of data transformation.

**Fig 1:**
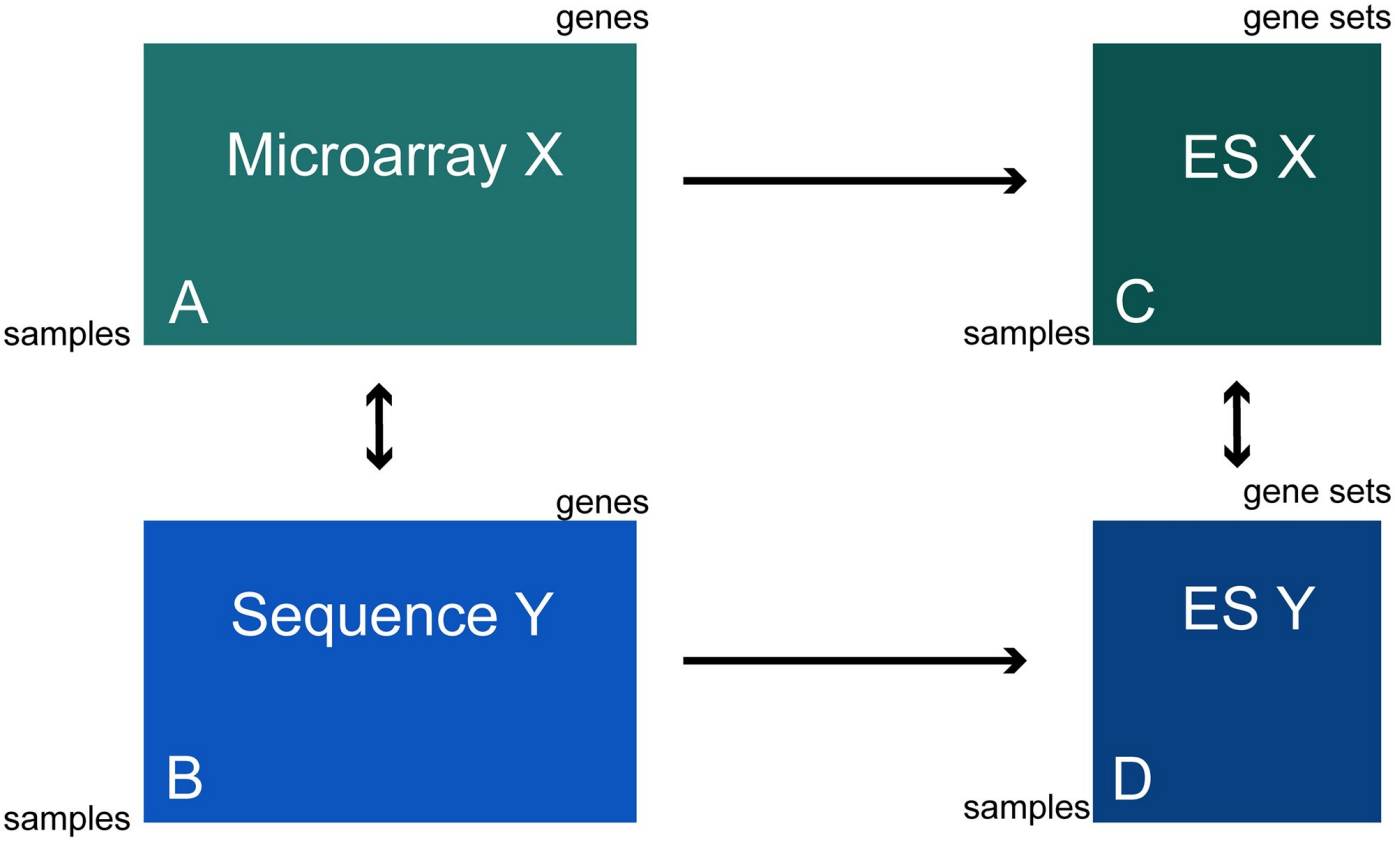
The basic setup of datasets that are compared in this study.

Fig 1 depicts the different types of data blocks we work with. We identify 4 different blocks; A through D. Because we are interested in whether or not we can improve the correspondence between the two platforms we only focus on differences between A and B on the one hand and C and D on the other hand.

We implemented the enrichment score according to [11] and applied the transformation to three cases of publicly available microarray and RNA-Seq datasets of the same set of samples. The first set of samples were obtained from the Cancer Cell Line Encyclopedia (CCLE) [12] which has a large overlap in samples measured on both microarray and RNA-Seq. The second dataset resulted from a study on activated T-cells in humans [5] and was much smaller. We will show that the enrichment scores increase the similarities between the two platforms compared to the original gene (raw) expression values and characterize the role of the gene sets. To demonstrate that biological information is retained by using this gene set transformation we used a third dataset from Thompson et al. [7]. We developed a logistic regression model using the enrichment scores based on the microarray data (TCGA) to predict different subtypes of breast cancer. The sequence data-set contained overlapping samples but also other (breast cancer) samples. Using the model based on microarray data we show that meta-analysis across platforms becomes possible.

## Materials and methods

In this paper we use 3 different datasets containing data from both the microarray and sequencing platforms. Below we describe the datasets. We included a schematic overview of overlapping samples between the two platforms for every dataset respectively in the supplemental figures S1, S2 and S3 Figs.

### Dataset 1, cancer cell line data (CCLE)

Two publicly available datasets of expression data from cancer cell lines covering 22 different histologies have been obtained from the Cancer Cell Line Encyclopedia (CCLE) consortium [12]. The expression values were determined by Affymetrix U133+2 microarrays and by RNA-Seq. We used the Robust Multi-array Average (RMA) normalized microarray data and raw read count (transcriptomic) values from RNA-Seq. Both were downloaded from the Broad Institute website [13]. To evaluate if inter-species comparison is feasible based on the enrichment score we also downloaded Transcript Per Million (TPM) normalized sequence data for the same cancer cell lines from this website.

The obtained microarray dataset from CCLE was annotated with HUGO gene symbols and Entrez IDs while the RNA-Seq dataset was annotated with HUGO gene symbols and Ensembl IDs. Although both datasets shared the HUGO gene symbols these were not unique in the datasets and were therefore discarded as the primary means of matching genes between datasets. We used the biomaRt R package [14, 15] to create an annotation table that maps the Ensembl IDs of the RNA-Seq dataset to the Entrez format. We first removed all genes from the datasets for which no mapping between formats could be obtained. Due to differences in gene definition between the Entrez and Ensembl format multiple genes from one dataset could in some cases be mapped to one gene of the other dataset. In such cases we used the HUGO gene symbol annotation column available in both datasets to determine which of the duplicate results should be matched. If gene mapping remained inconclusive after these steps, the genes in question were removed from both datasets. After removing samples that were in only one of the datasets we arrived at two datasets of 970 samples for which the expression values of 17,415 genes were determined.

### Dataset 2, in vivo data

We obtained an in vivo dataset from a comparative transcriptomics study on activated CD4^+^ human T-cells [5] to test the transformation in a second example, biologically very different from the CCLE dataset. The data from this study encompasses expression values for samples obtained at six different time points from one individual. Each sample had a replicate resulting in a total of 12 samples for which both Affymetrix GeneChip HT HG-U133+PM microarray and RNA-Seq data are available. We used the RMA normalized microarray dataset and the raw RNA-Seq counts data. Only those genes for which values were established on both platforms were maintained. This resulted in two comparable datasets of 18,304 gene expression values measured for all 12 samples. The correlation between the repeats is very high (0.996 plus) for each platform.

### Dataset 3, breast cancer data (TCGA)

The breast cancer data is well described in the paper by Thompson et al. [7] and consisted of samples of different types of breast cancer from The Cancer Genome Atlas (TCGA). Both microarray and RNA-seq were used to measure gene expression. The microarray data consisted of 577 samples with 4 different subtypes of breast cancer and non-carcinoma samples. The sequence data contained the same 577 samples and contained an additional 379 samples.

### Gene set collections

The Molecular Signatures Database v6.0 (MSigDB) [5] from Broad Institute provides 8 publicly available gene set collections that cover a range of biological functions. The number of genes and the number of sets in the different gene set collections from the database vary widely covering from 4,386 to 30,012 unique coding and non-coding gene definitions and varying from 50 to 4,872 sets in a collection. Individual gene sets can vary from 5 to 2,940 genes (see Table 1). We calculated the enrichment scores for both the microarray and the RNA-Seq data for all the different datasets with gene set collections H, C6 and C7 of MSigDB v6.2. These gene set collections are expected to be in line with the biological origin of our datasets. We used collection C6 that represents ‘biological processes that are commonly dysregulated in cancer’ for the cancer cell line dataset (dataset 1). This collection consists of 189 individual gene sets and covers a total of 11,250 unique genes. As a result, the data is transformed/compressed from 17,415 genes to 189 enrichment score variables.

**Table 1:** The composition of the eight gene set collections of the MSigDB v6.0. Shown are the number of sets, the smallest, largest and average set size by number of genes. The total number of genes and the number of unique genes. The last column represents the highest number of sets a single gene was encountered in.

Name	# gene Sets	Min set size	Max Set size	Mean Set size	Total # genes	Unique # genes	Max occurrence
H	50	32	200	146	7,324	4,386	10
C1	326	5	948	93	30,288	30,012	3
C2	4,731	5	1,972	93	441,206	21,086	285
C3	836	5	2,940	237	198,275	14,085	232
C4	858	10	838	106	91,309	10,062	90
C5	5,917	10	1,984	117	689,570	17,106	659
C6	189	10	481	166	31,319	11,250	28
C7	4,872	90	200	195	948,254	20,652	284

For the 12 samples in the in vivo dataset (dataset 2) in which the immune system is perturbed we used the C7 gene set collection to transform. The C7 collection encompasses manually curated cell states and perturbations from immunology studies. The C7 collection is composed of 4,872 gene sets and will thus reduce the number of variables of this dataset (2) from 18,304 genes to 4,872 enrichment score variables.

As a control we use the hallmark (H) collection which is, with only 50 gene sets, the smallest of the available collections, see Table 1. This collection is constructed from different gene sets from the other collections ‘to represent well defined biological states and processes’. From Table 1 it is clear that the different collections use the genes in different configurations but also that every collections has gene sets that contain genes that are used in multiple gene sets (e.g. C5, up to 659 times a use of the same gene in as many gene sets). Conversely, gene set C1 has a maximum occurrence of a gene in only 3 different gene sets and therefore has the most ‘distinctive’ gene sets.

### Gene set transformation

Gene set enrichment scores were determined as proposed by [11]. The enrichment score *ES(H)* for gene set *H* is calculated for each sample. First, the gene expression values *G* of all *R* genes in that sample are sorted from high to low to determine the rank *r (= 1…R)* for each gene. Thus, for the gene with the largest expression value *r = 1* while *r = R* for the lowest gene expression value. *ES(H)* is calculated as the cumulative sum of the differences between the cumulative probability sum (of all genes in the sample) that the genes belong to gene set *H (P*_*H*_*)* minus the cumulative probability sum that genes do not belong the gene set *H* (*P*_*NH*_) (Eqs 1–3).

In these equations the counting variable *r*, representing the rank, goes from 1 to the total number of genes *R*. In Eq 2, the sum is only taken over the genes up to rank *r* that belong gene set *H*, where *I* is an indicator variable meaning *I(G*_*i*_*∈H)* equals 1 when gene *G*_*i*_ is a member of gene set *H*, while it is 0 otherwise. In Eq 3, the sum is taken over the first *r* genes that do not belong to gene set *H*, *I(G*_*i*_*∉H)*. *R*_*H*_ represents the number of genes that are in gene set *H*. The stabilizing power *α* was left at a value of 0.25 as suggested in [11].

Both Eqs 2 and 3 go from 0 to 1 in *R*_*H*_ and (*R-R*_*H*_) steps respectively. If the average rank of genes belonging to gene set *H* is high (or low), then there is an early (or late) fast increase in *P*_*H*_. If the genes in gene set *H* have no preference and are randomly spread over the ranked genes *P*_*H*_ closely follows *P*_*NH*_.

$$ES(H)=\sum_{r=1}^{R}\left(P_{H}(H,r)-P_{NH}(H,r)\right)$$ (1)

$$P_{H}(H,r)=\sum_{i=1}^{r}I(G_{i}\;\epsilon \;H)\frac{i^{\alpha }}{\sum_{i=1}^{R}I(G_{i}\epsilon H)i^{\alpha }}$$ (2)

$$P_{NH}(H,r)=\sum_{i=1}^{r}I(G_{i}\notin H)\frac{1}{R-R_{H}}$$ (3)

**Fig 2:**
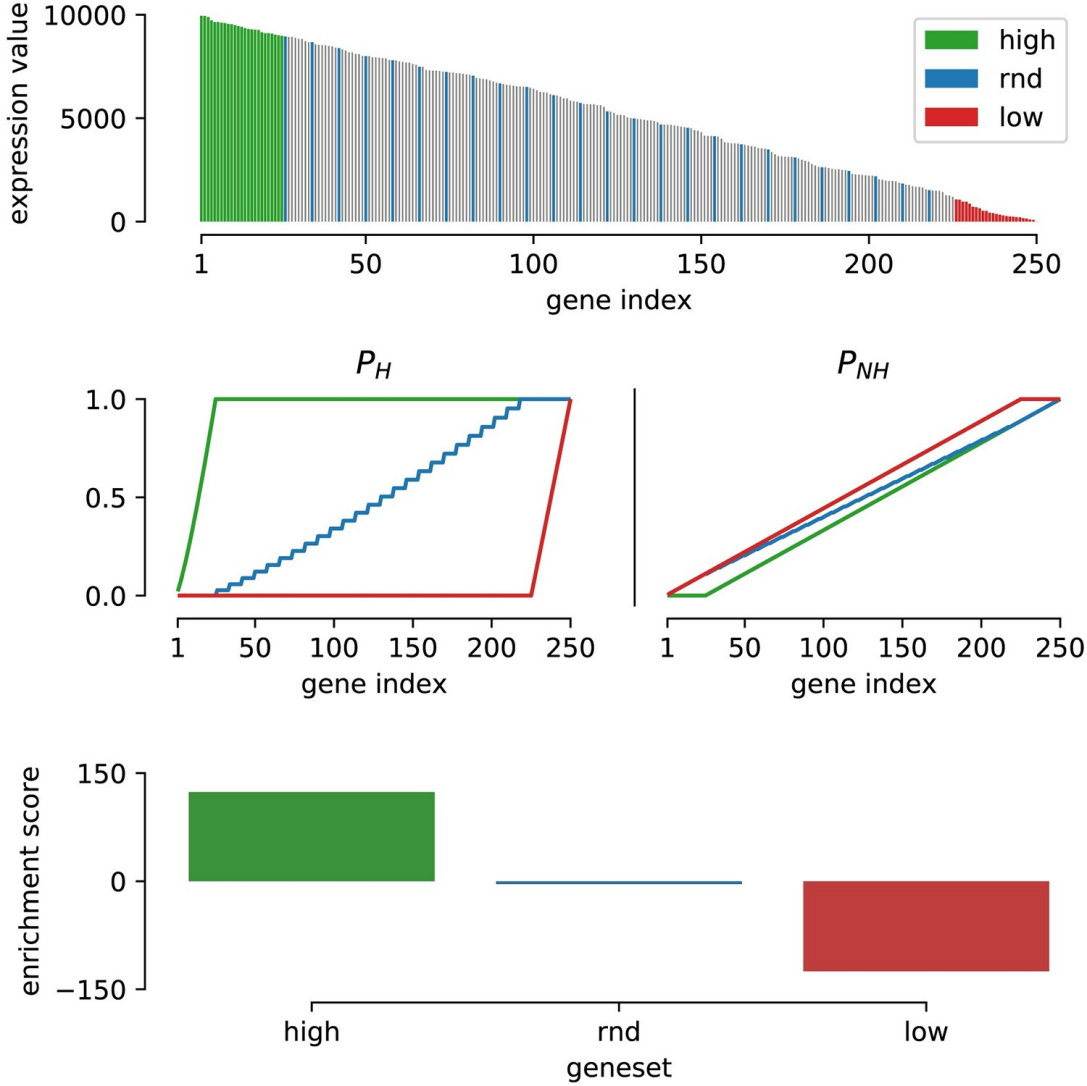
Demonstration of the enrichment score calculation with a simulated dataset. Top: sorted expression levels of 250 genes in which coloring represents gene set membership. Middle: Left, the calculated *P*_*H*_ values for each gene set. Right, the *P*_*NH*_ values for each gene set. Bottom: The resulting enrichment scores for each of the three gene sets.

Fig 2 shows the application of the transformation on artificial data in which 250 genes and 3 gene sets (one with high ranks, one with low ranks and one with no preferred (rnd) ranks) were simulated. The top figure represents the ranked expression scores of the sample in which the coloring of the bars denotes their membership to any of the three artificial gene sets (green, blue, red). The grey colored bars represent genes that are not present in any of the three gene sets. The second plot shows the calculated values for P_H_ (solid) and P_NH_ colored in correspondence to the different gene sets. The resulting enrichment score for each gene set is the resulting area described by the summed difference between the two lines. For gene sets with members randomly dispersed throughout the ranked expression landscape of the sample (blue), *P*_*H*_ and *P*_*NH*_ will closely track each other leading to an enrichment score close to zero. In contrast, gene sets of which the members show a clear tendency to aggregate to high (green) or low (red) expression values result in high and low enrichment scores respectively. With this approach genes in a single sample can be ‘scored’ on many different gene sets and can be tested for differential analysis later whereas in the ‘normal’ (enrichment analysis) approach the gene set scores (pathway enrichment scores) are determined based on group averages.

### Congruence between the two platforms

Microarray results are influenced by the hybridization efficiency of the probes and not necessarily proportional to the molar concentrations of mRNA in the samples while the RNA-seq read counts are influenced for example by the sequencing depth of the sample. Both platforms have their own biases. Discrepancies between the two transcriptomics platforms can clearly be visualized in a scatterplot when the two axes represent the microarray values and the RNA-Seq values respectively of the same sample. In such a plot ideally, all points would lie on a line with intercept 0 and slope 1 when equal gene expression levels are found by both platforms. Because the platforms generate different gene expression levels, possibly of different magnitudes, the intercept and slope are expected to be different from 0 and 1 respectively. To quantify the difference between the two platforms we use the Spearman (rank) correlation and the residual variance (***var(E)***) value. This residual variance is calculated by performing a Principal Component Analysis (PCA) on the combined RNA-seq and microarray data for the two samples to a single data matrix. At most 2 principal components can be obtained from which the first (PC1) explains the most variance and if all points lie on a straight line, captures 100% of all variation. The remainder (PC2) therefore explains 100% minus the explained PC1 variance and represents the deviation from the ideal situation The larger ***var(E)***, the more points are deviating from the straight line, the higher the dispersion and/or nonlinearity between the two platforms. Additionally, we also determine the similarity between the original platform datasets and the gene set compressed datasets as a whole by means of the modified RV coefficient [16]. The modified RV coefficient is a matrix correlation measure based on the configuration of the samples, e.g. if grouping of samples is similar in two matrices, then their modified RV coefficient is high (close to 1). The modified RV coefficient was calculated after mean centering and scaling of unit variance for each column in both datasets.

### Gene set permutations

To assess whether the gene set collections capture real biological information by an appropriate selection of genes into the gene sets, we performed a permutation test. The permutation test should answer whether the defined gene sets are a better collection of genes to perform a specific task than any random selection of genes. We permute the original gene set collection C6 (oncogenic gene sets) by randomly selecting genes from the whole pool of genes in the collection for every gene set. Similar to the original gene sets, a permuted gene set can contain a gene only once. This effectively eliminates the biological relevance of the gene sets while leaving the specific structure i.e., the number of genes per set and the total number of genes in the collection, intact. By removing the relationship between the selected genes in a gene set, we obtain enrichment score values unrelated to biological events. We will test whether the originally obtained enrichment score values are different from the ones obtained after permutation (H_0_). After every permutation we recalculate the resulting gene set enrichment scores on a sample for both platforms and determine the Spearman correlations between the resulting vectors. This is done for all 970 samples of the CCLE datasets with 100 permutations. We thereby obtain a H_0_ distribution of 97,000 values against which we compare the results that were found using the original gene set collection.

## Results and discussion

Using the three datasets we will demonstrate the increase in similarity when gene set transformation is applied. Next we attempt to investigate why this approach works using permutations and a simpler rank score.

### Dataset 1, cancer cell line data (CCLE)

**Fig 3:**
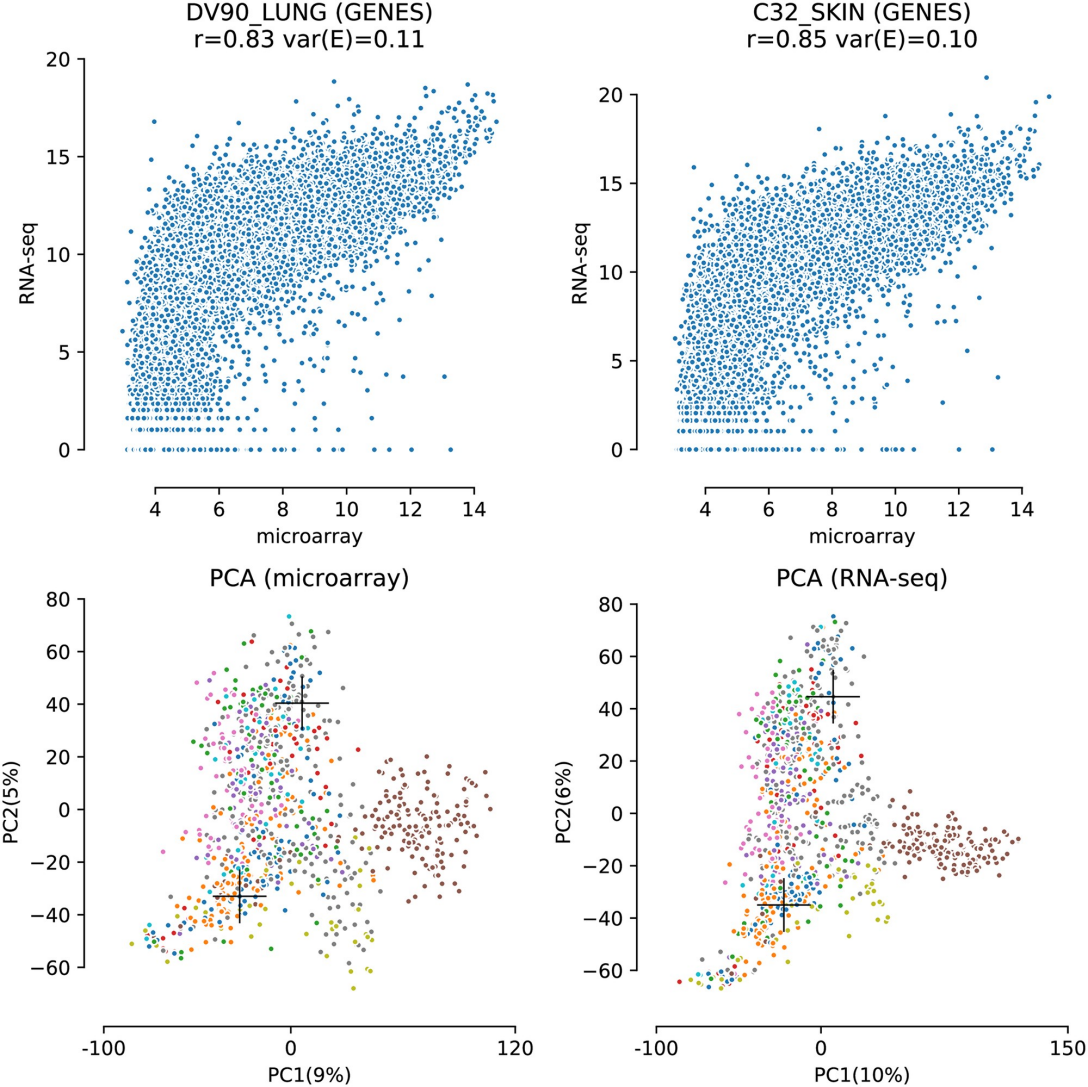
Top; two scatterplots of samples from the CCLE datasets, after log2 transformation, that show concordance between raw gene expression levels as determined by microarray and RNA-Seq. Spearman correlation coefficients are given for each plot. The clear horizontal lines near the bottom are an artifact of the nature of RNA-Seq data which are integer counts in contrast to the analog microarray values. Bottom; the first two principal components showing the distribution of samples for each dataset. Coloring of the samples is histology based and the crosshairs denote the two samples used for the scatterplots in the top figure.

Correlations between microarray and RNA-Seq have been studied extensively [5, 6], however, correlation on its own might not be sufficient to capture platform concordance. In Fig 3, in the top row, two scatterplots are shown for two selected samples from the CCLE dataset. From these figures it is apparent that the two platforms do not provide the same information as the points clearly deviate from the diagonal. Note that the scales of the two platforms are naturally different. Although the Spearman correlation values above each plot (signified by rho) are high, a large amount of dispersion is still observed in each plot.

In conjunction to the Spearman correlation we scored concordance between platforms by residual variance, ***var(E)*** (see section “Congruence between the two platforms”). This value represents the amount of dispersion and nonlinearity and is given above each plot as ***var(E)*** as a fraction of the total variation.

To gain insight in the relationships between all 970 samples in the CCLE dataset (dataset 1) we performed a PCA of the microarray and RNA-Seq dataset separately. The resulting score plots are provided at the bottom row of Fig 3 in which the samples are colored by their histology. These plots clearly indicate grouping related to the different histology. Although the PCA score plots are similar some small differences can be observed (e.g. the brown group on the right is more spread out in the microarray score plot). The two samples for which scatter plots (top) are shown are marked with crosses in the score plots (bottom). The distance between the two samples in these score plots indicates that they are very different in nature yet their scatter plots appear very similar.

### Gene set transformed data

**Fig 4:**
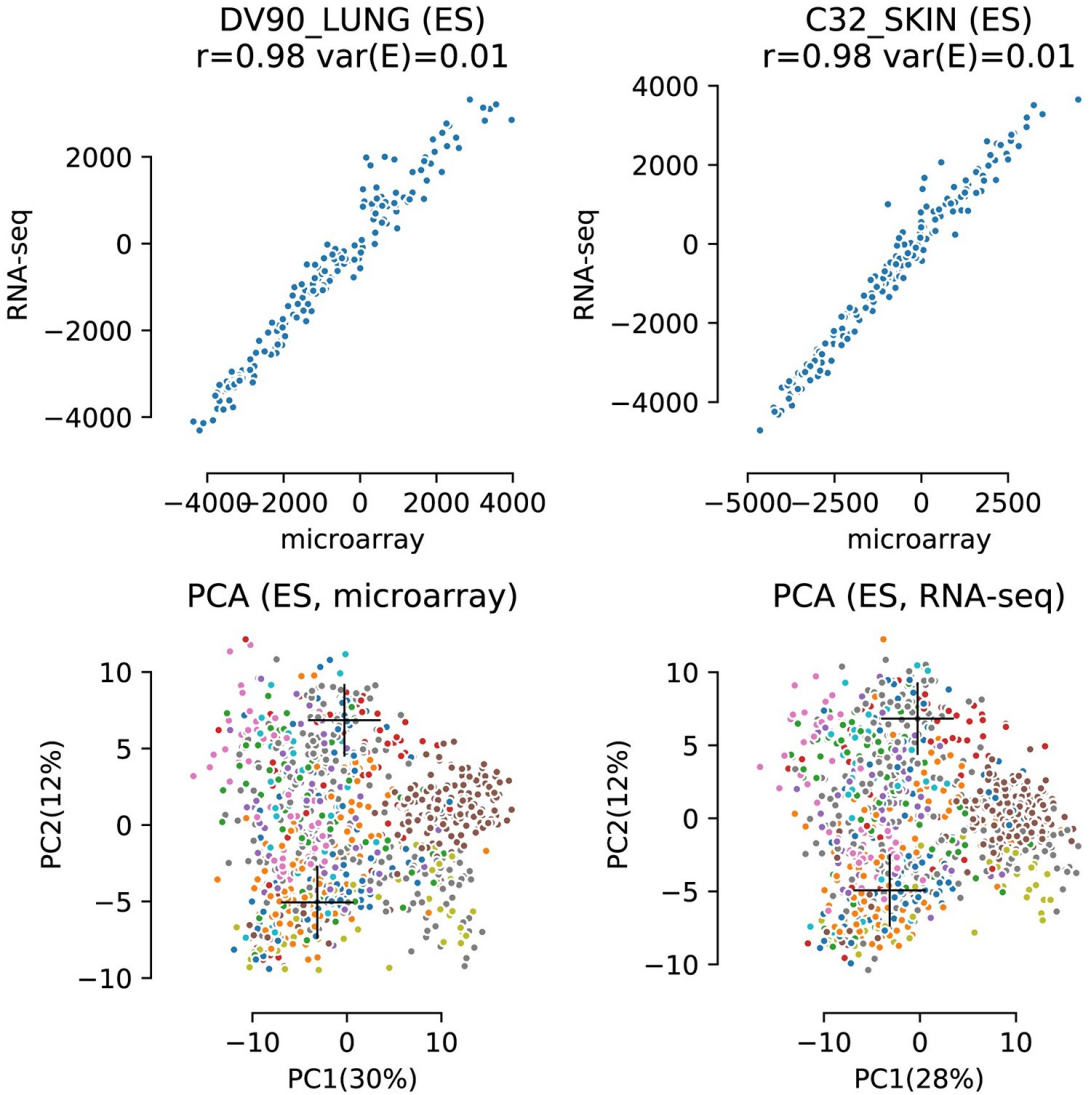
Top; two scatterplots of samples from the CCLE data after conversion to gene set based enrichment scores. Spearman correlations (rho) are given for each plot. Bottom; the first two principal components (PCA on ES values) showing the distribution of samples for each dataset. Coloring of the samples is based on histology and the crosses denote the two samples used for the scatterplots.

In our research we calculated enrichment scores for the 189 gene sets in the C6 (oncogenic) gene set collection. The scores were determined for all samples of both the microarray and RNA-Seq datasets individually, as illustrated in Fig 4. Data compression resulting from the approach is apparent by the reduced number of data points in comparison to the gene based data shown in Fig 3. The number of variables reduced from gene expression levels for all genes that were present in both datasets (17,415) to the number of gene sets in the gene set collection C6, 189. It should be noted as well that the gene expression levels in Fig 3 were log transformed while the enrichment scores are not. Ranks and consequently enrichment scores are invariant to prior log transformation of the raw data.

The scatterplots in Fig 4 (top) clearly show that concordance between both platforms has improved considerably when the data is studied on a gene set level. This notion is confirmed by the respective Spearman correlation coefficient and the ***var(E)*** values (above each plot). The bottom two plots of Fig 4 show PCA score plots for both microarray and RNA-Seq datasets after gene set transformation. One could argue that because of the smaller number of datapoints the comparison based on correlation values is biased. We therefore compared the similarity of the two PCA score plots in Fig 4 to the similarity of the pair from Fig 3 (bottom) by means of a matrix correlation value. The modified RV coefficient of the pair in Fig 3 before gene set transformation was 0.915. This coefficient increased to 0.948 for the PCA scores plots after transformation and indicates more similarity between microarray and RNA-Seq datasets when they have been transformed to gene set enrichment scores.

Transcripts per Million (TPM) normalization, in which the transcript count is corrected for by the gene length can clearly influence the rank of the genes and therefore the enrichment score. We used the TPM normalized sequence data from dataset 1 that overlapped with our original sequence data by 916 samples to evaluate this effect and recalculated the enrichment scores for the different gene sets (H,C6 and C7). The average correlation between the microarray based enrichment scores and the sequence based enrichment scores remained very high (0.98–0.99) and indicated that this transformation could be used for inter-species comparison.

**Fig 5:**
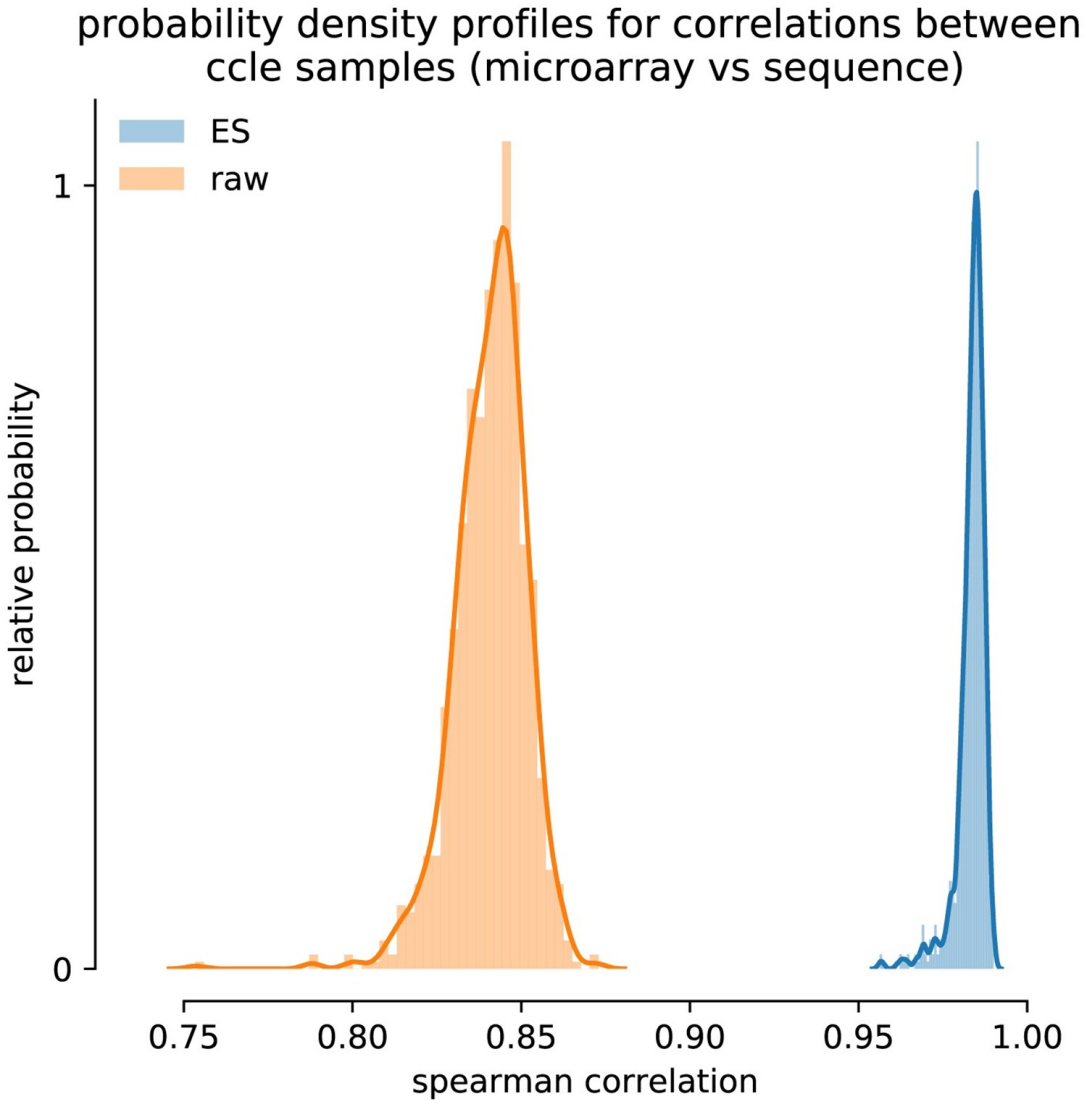
The distribution of Spearman correlations found between microarray and RNA-Seq data before (orange: Mean correlation = 0.841) and after (blue: Mean correlation = 0.982) gene set enrichment analysis based on gene set collection C6 (see Table 1).

The increased consensus between microarray and RNA-Seq at gene set level was found to be systematic across all 970 samples, as can be seen in Fig 5. The histograms represent the distribution of pairwise Spearman correlation coefficients between microarray and RNA-Seq sample data. The gene based data (orange) resulted in an average Spearman correlation coefficient of 0.841 while for the gene set transformed data (blue) it resulted in a much higher correlation of 0.982.

### Dataset 2, in vivo data

**Fig 6:**
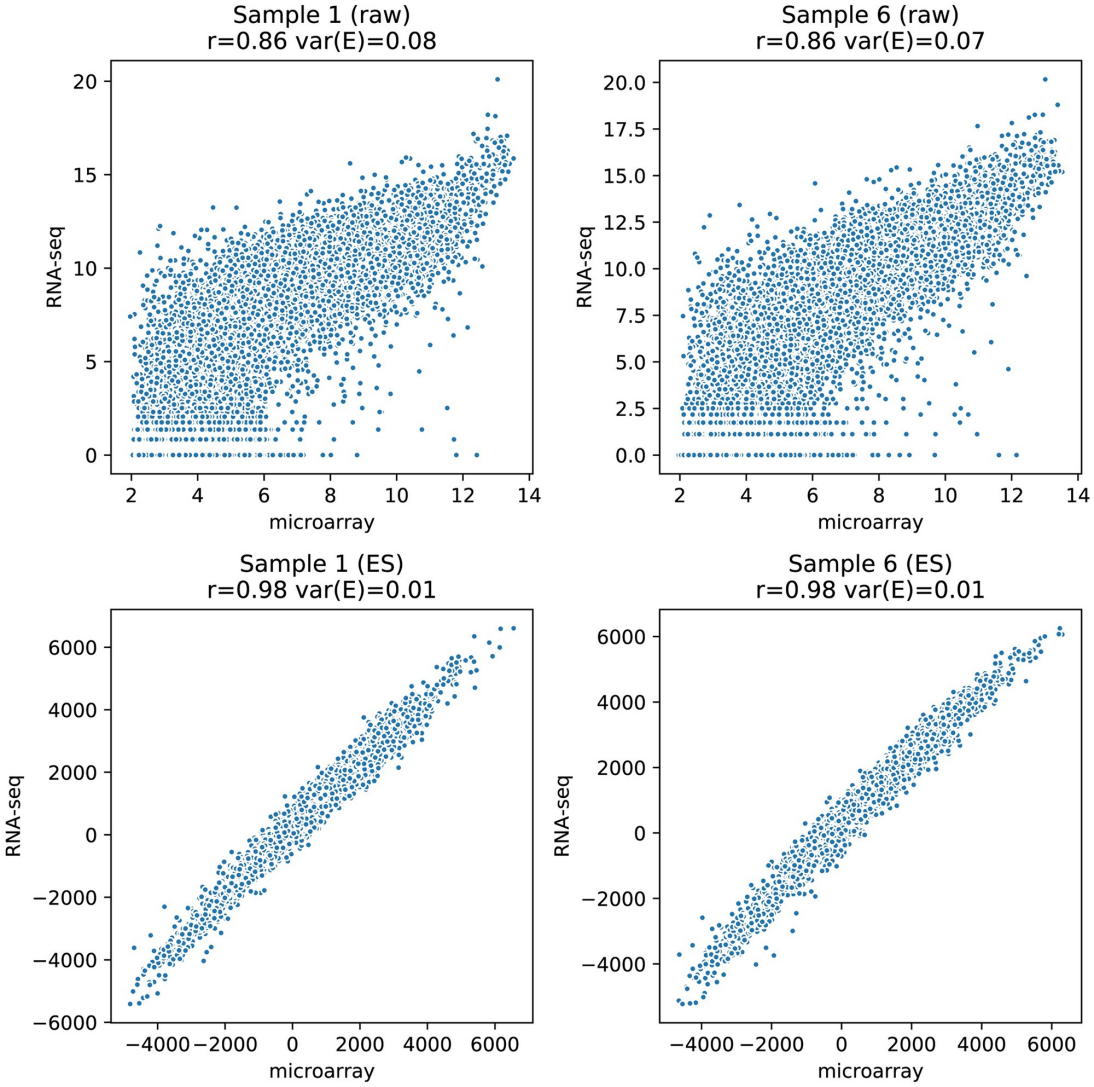
Scatterplots of two samples from dataset 2 that show gene-based expression levels as determined by microarray and RNA-Seq(top) and the result after enrichment scores were calculated for the 4872 sets of gene set collection C7 (bottom). The corresponding Spearman correlation coefficient values and the var(E) values are given at the top of each plot.

Next to the CCLE dataset we also tested the approach on a smaller (n = 12) dataset on gene expression in activated CD4^+^ human T-cells. Transformation of this dataset however, was performed with gene set collection C7 which reduced the number of variables from 18,304 genes to 4,872 gene sets. Fig 6 shows scatter plots for 2 (not replicates) of the 12 samples before (top) and after (bottom) gene set transformation. These plots further strengthen the notion of an increased concordance between the two platforms when comparing enrichment scores instead of the gene expression values. This improvement is again reflected by the Spearman correlation coefficient values and ***var(E)*** values above each plot. Again, results were systematic for all 12 samples in the dataset. Spearman correlation coefficient averages between microarray and RNA-Seq were 0.864 and 0.982 for gene based and gene set transformed data respectively. The correlation between the repeats remained very high (0.999+) for each platform.

### Dataset 3, breast cancer data (TCGA)

To demonstrate that the transformation retains biological information which can even be used in a meta-analysis context we used the data which was also used by Thompson et al [7]. The dataset contained samples originating from 4 subtypes of breast cancer or non-carcinoma cell lines (Basal, Her2, LumA, LumB and Normal). A schematic of the dataset is included in the supplemental document (see S3 and S4 Figs). Both microarray data (X_*MA*_) and sequence data (X_*SEQ*_) contained the same 577 cell-lines but the sequence data also contained samples from 379 additional cell-lines.

### Using the same cell-line samples

The microarray data was transformed to enrichment scores (ES_*MA_(H*,*C6*,*C7)*_) for gene sets H, C6 and C7 and based on these scores predictive logistic (cross-validated) models were created to predict the subtype. The choice of test and training sets during cross-validation was dependent on a random selection method. Similar to Thompson we repeated the modelling process 10 times, each with different training/test sets. Table 2 shows the confusion matrix of the combined 10 models for the training set (C6 enrichment scores). The average accuracy is high (0.95).

**Table 2:** Confusion table for the model predictions based on the microarray enrichment scores (ES_MA_C6_).

	**Basal**	**Her2**	**LumA**	**LumB**	**Normal**
**Basal**	910	0	20	0	0
**Her2**	0	420	0	30	0
**LumA**	20	10	2630	20	30
**LumB**	0	0	100	960	0
**Normal**	0	0	31	0	589

To assess if meta-analysis is possible we used the models that were created before, based on the microarray enrichment scores (ES_*MA*_), to predict the breast cancer subtypes of the 577 overlapping samples in ES_*SEQ_C6*._ Table 3 shows the resulting confusion table and with an average accuracy of 0.32 the predictive quality is very poor.

**Table 3:** Confusion table for the model predictions based on sequence array enrichment scores (ES_SEQ_C6_) for overlapping samples.

	**Basal**	**Her2**	**LumA**	**LumB**	**Normal**
**Basal**	0	925	0	5	0
**Her2**	0	450	0	0	0
**LumA**	0	1448	718	544	0
**LumB**	0	395	0	665	0
**Normal**	0	76	540	4	0

**Fig 7:**
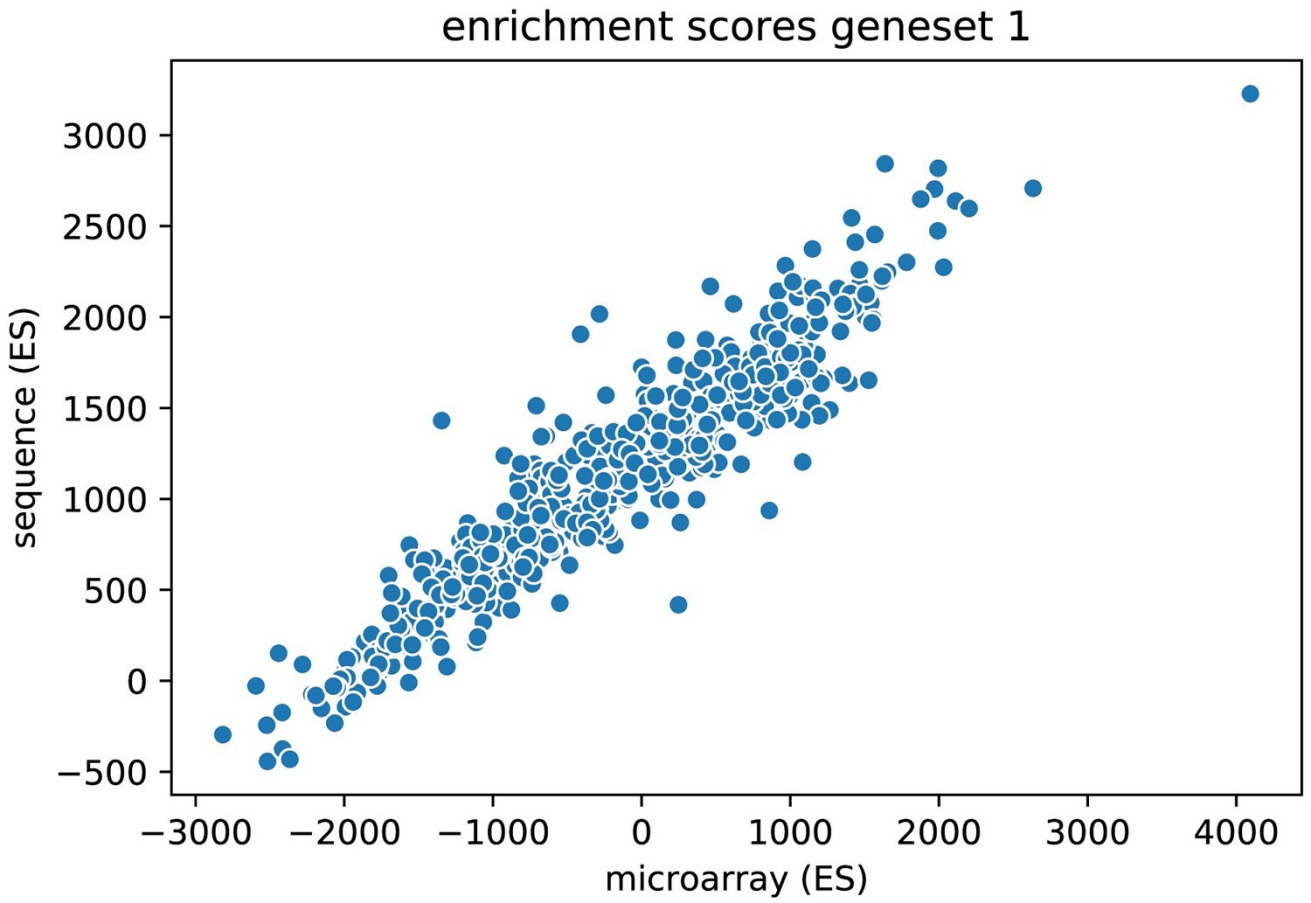
An offset between enrichment scores (for gene set 1, from C6) based on microarray data and sequence data.

Closer inspection of the difference between the microarray based (ES_*MA_C6*_) and sequence based (ES_*SEQ_C6*_) enrichment scores revealed that there was a noticeable offset between the two types of gene set scores (see Fig 7). In Fig 7 the ES_*MA_C6*_ scores for all samples are plotted against the overlapping samples in ES_*SEQ_C6*_. It can be seen that for the lowest value the ES_*MA_C6*_ score is -3000 while for that same sample the lowest ES_*SEQ_C6*_ score is -500. In the Supplemental document we investigate the origin of this offset and conclude that this is caused by many values having the same rank in the data.

This phenomenon can commonly be observed in sequence data where the read count for many genes is zero. As we demonstrated in the Supplemental document (S1 Text), having many low values (e.g. missing/zeros) in the data gives a positive (non-uniform) shift to the enrichment scores. Because it could well be that specific breast cancer types show more zeros in their sequenced data than other types we cannot ignore this non-uniform nature. We therefore used a Procrustes analysis [17] to align the sequence based (ES_*SEQ_C6*_) and microarray based enrichment scores (ES_*MA_C6*_) for the overlapping samples. The Procrustes transformation consists of three steps, translation, scaling and orthogonal rotation, to align the data in two datasets.

The resulting transformation function (T) was applied to all the enrichment scores in ES_*SEQ_C6*_ resulting in ES_*T_SEQ_C6*_. The original models based on microarray data were again used to predict the subtype of breast cancer (see Table 4). This time however, an accuracy of 0.85 was obtained, indicating that the model regained its predictive capability.

**Table 4:** Confusion table for the model prediction based on sequence array enrichment scores (ES_T_SEQ_C6_) for overlapping samples after Procrustes transformation.

	**Basal**	**Her2**	**LumA**	**LumB**	**Normal**
**Basal**	890	20	10	0	10
**Her2**	10	332	50	58	0
**LumA**	0	20	2605	65	20
**LumB**	0	17	255	788	0
**Normal**	30	0	144	0	446

The overall prediction accuracy for all (n = 956) samples in the sequence dataset (ES_*T_SEQ_C6*_) was 0.85.

### Using different cell line samples

The overlap between the two platforms was large (n = 577 samples) and a situation can be envisioned in which there is no overlap at all. To test our approach in an extreme case when there is no overlap of samples at all between the two platforms we used the Procrustes transformation to align the 5 group means of the 577 samples in ES_*MA*_ and the 5 group means of the 379 non-overlapping samples in ES_*SEQ*_.

The resulting transformation function (T2) was applied to all the data in ES_*SEQ_C6*_ and the enrichment scores for the sequence data were updated (ES_*T2_SEQ_C6*_). The prediction accuracy of the original models using these updated C6 scores was 0.76 which furthermore highlighted the predictive capability of the model.

These steps were repeated for the models based on the H and C7 gene set scores. The accuracy scores are shown in Table 5. The results in the second row (ES_*T_SEQ_*.._) can be compared to results in the original paper of Thompson [7]; their best was 0.85.

**Table 5:** Accuracy scores for prediction of the models using the different gene sets scores.

Input	H	C6	C7
**ES**_***MA_*..**_	0.91	0.95	1
**ES**_***T_SEQ_*..**_	0.85	0.85	0.91
**ES**_***T2_SEQ_*..**_	0.71	0.76	0.64

### Assessment of gene set enrichment scores for improving platform concordance

To assess why and how the transformation of gene expression values into gene set enrichment values improve the concordance between the microarray and RNA-seq measurements we first will study whether the improved correlation is due to ranking, or whether it is the result of averaging over a set of related genes.

### Using ranks instead of quantitative values

**Fig 8:**
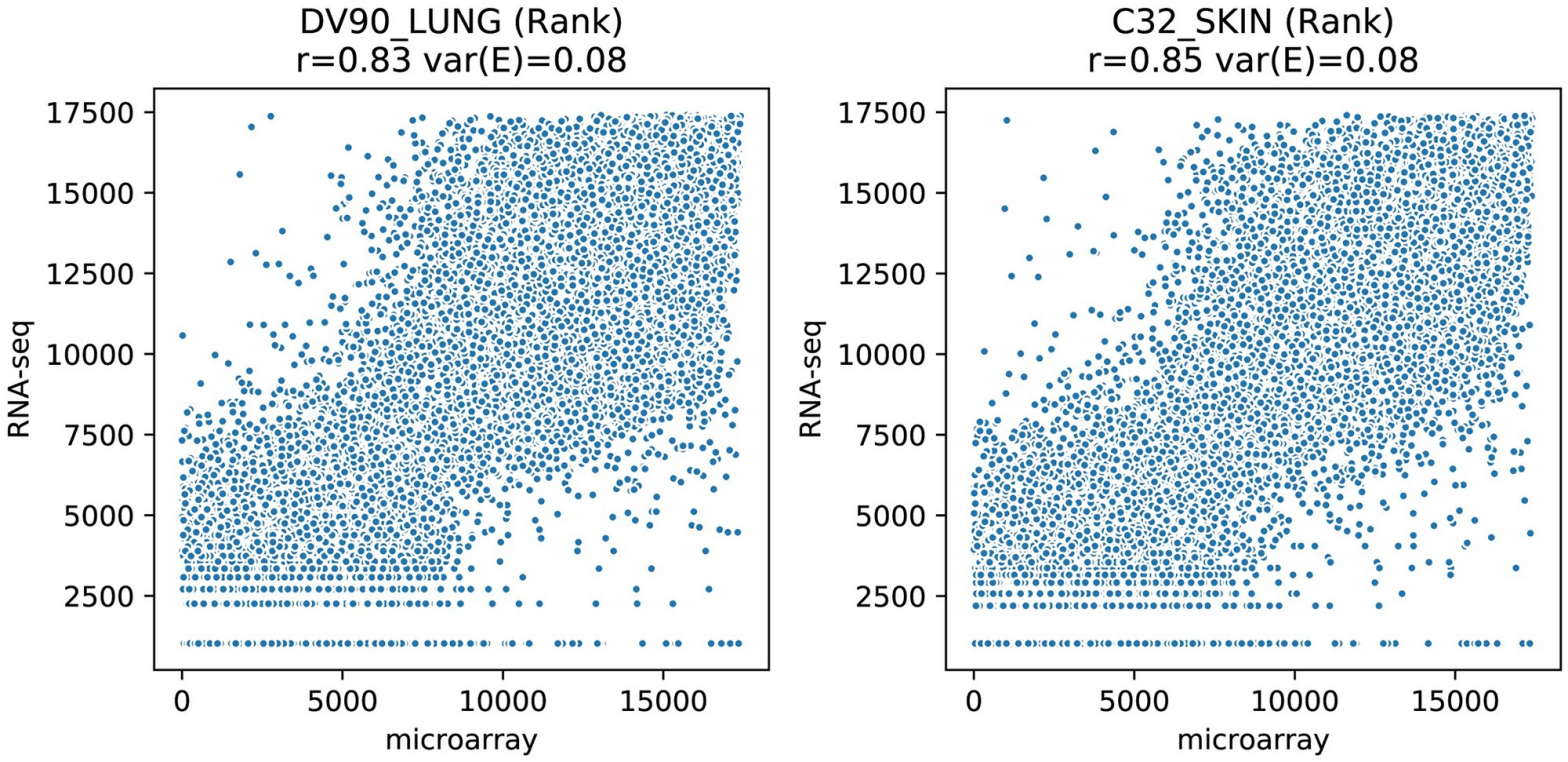
Comparison between ranked gene expression scores as established by microarray and RNA-Seq (from CCLE, dataset 1). The samples are identical to the samples shown in Figs 3 and 4. Because the quantitative values for many genes is equal in RNA-seq they are assigned the same rank which causes the horizontal lines in the plot.

We studied whether the increased concordances between microarray and RNA-Seq could solely be attributed to the transformation of quantitative values to ranks in the computation of the gene set enrichment scores. To do so, we tested the effect of the ranking itself on the same samples (from CCLE, dataset 1) that were used before in the scatter plots of Figs 3 and 4. Scatter plots resulting from a simple ranking are shown in Fig 8. The Spearman correlations are identical to the values shown in Fig 2 because the Spearman correlation is already calculated on ranks by definition. The ***var(E)*** value shows a slight improvement as a result of the improved linearity (from 0.11 and 0.1 to 0.08). This improvement is, however, mostly obscured by the evident dispersion and is still much worse compared to the ***var(E)*** values of 0.01 that was found after gene set transformation, as shown in Fig 4.

### Is improvement caused by averaging or by biological information in gene sets

**Fig 9:**
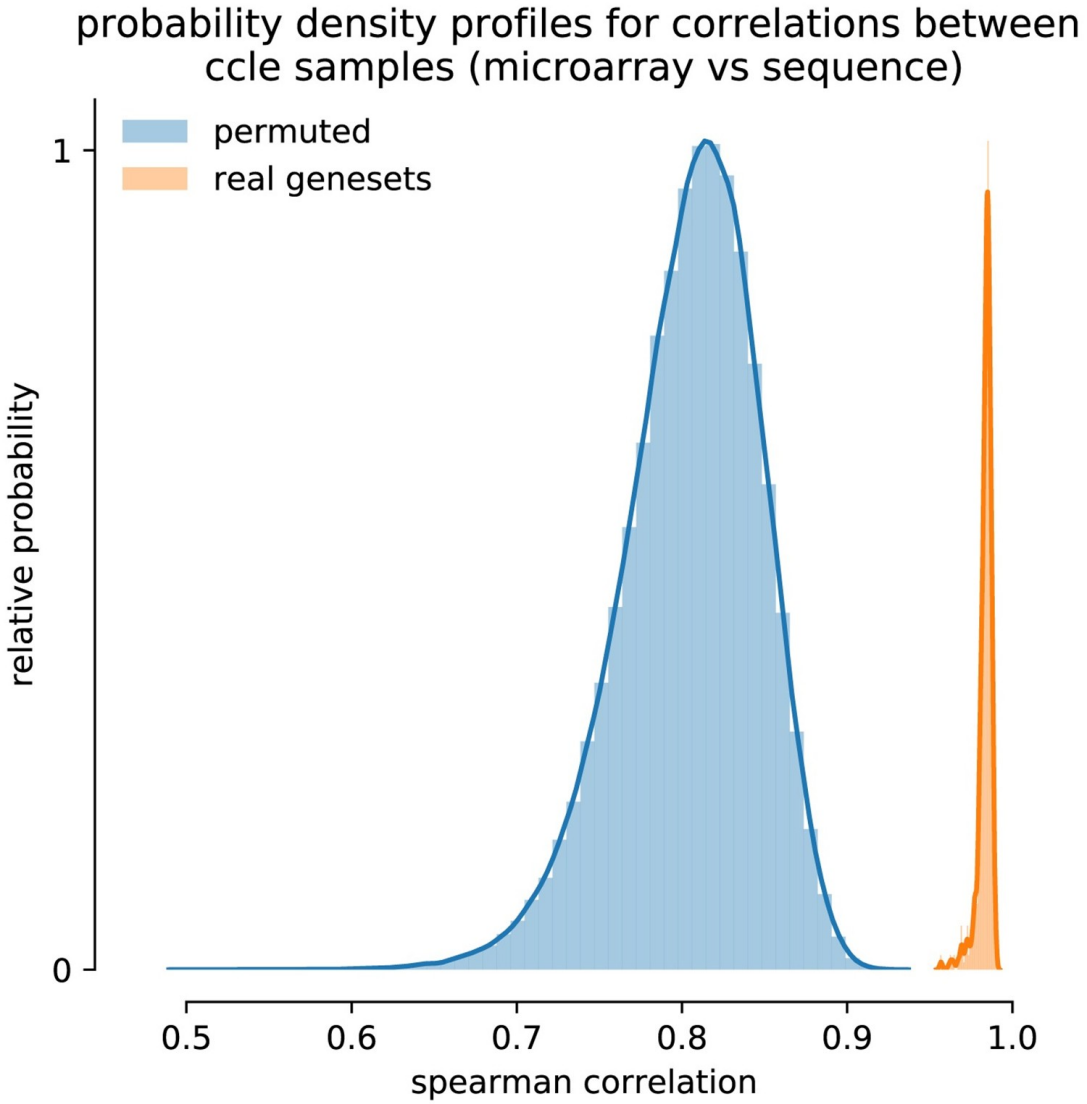
Obtained spearman correlations between the two enrichment score vectors computed for every sample with the RNA-Seq and microarray data. The blue histogram (left) shows the results after permuting the gene set (mean correlation = 0.804). The orange histogram (right) represents results obtained with original gene set collection (mean correlation = 0.982).

Alternatively, to the ranking of values we postulated that the reduction to gene sets causes an averaging effect that equalizes extreme individual gene responses and filters technological noise. If this is the case, we expect to find similar results by utilizing a randomly selected gene set without any prior biological context. To test this theory, we performed a permutation analysis in which gene sets were generated by randomly replacing genes with any other gene occurring in the total of combined gene sets collection. The observed Spearman correlation distributions for the resulting gene set scores are shown in Fig 9. In this figure the blue and orange histograms represent the results obtained with a permuted and the original gene set collection (C6) respectively. The permuted gene sets show an average correlation of 0.804 which is mainly due to the averaging effect when comparing sets of genes instead of single genes. The average of 0.982 when using the real gene set definitions is significantly higher and the lack of overlap between these histograms is indicative that the obtained results are unique to the composition of the gene sets. As a result, we can state that the biological context within the collection is actively used to obtain the improved concordances.

To emphasize the fact that the gene sets are indeed not random we speculate that the median rank score should be a robust estimate for the gene set score. We tested its performance by transforming the entire CCLE dataset (dataset 1) using the median rank score only. Table 6 shows the Spearman correlation and ***var(E)*** results to indicate the similarity of the two platforms using both enrichment scores and the median rank score. Using the C6 gene set collection we obtained an average platform correlation over all samples of 0.973 with the median rank score, compared to 0.982 with the enrichment scores (Table 6). The ***var(E)*** was 0.02 with median rank scores and 0.01 for enrichment scores. According to these values the simpler median rank score performs significantly less (using Wilcoxon signed-rank test p-values) in terms of sample correlation and residual variance when compared to the enrichment score method but much better than the raw data which confirms that the median is a robust approximation for enriched gene sets.

**Table 6:** Average Spearman correlation and var(E) values between all samples of the microarray and RNA-Seq datasets before (Gene based) and after gene set transformation (using H, C6 and C7 gene set collections) using the Enrichment score and the median for transformation.

Method		Gene based	Gene set H	Gene set C6	Gene set C7
Enrichment score	Spearman ***var(E)***	0.841	0.972	**0.982**	0.973
		0.104	0.015	**0.011**	0.014
Median rank score	Spearman ***var(E)***	0.841	0.946	**0.973**	0.930
		0.104	0.025	**0.016**	0.026

### Selection of gene set collections

The selection of the oncogenic C6 gene set collection for the CCLE cancer data (dataset 1) and the immunologic gene sets in C7 for the in-vivo data set 2, with focus on human T-cells seemed straightforward but the question of which collection of gene sets would be optimal for a given dataset cannot be answered at this moment. The Broad Institute defined 8 different collections. Their appearances (in terms of number of gene sets, number of genes, sizes of gene-sets etc.) are so different that it is difficult to assess which property makes them behave different in the enrichment score calculation. This is emphasized by the results in Table 7 that shows the performance in terms of modified RV coefficients of the microarray and RNA-seq based enrichment scores (dataset 1) for the different gene set collections. The scores are clearly not that far apart and moreover, the H-collection gene sets seem to perform best.

**Table 7:** Modified RV coefficients of microarray and RNA-Seq datasets before and after gene set transformation using different gene set collections.

Dataset	Gene based	Gene set H	Gene set C6	Gene set C7
Modified RV	0.915	0.952	0.948	0.949

## Discussion and conclusion

In this paper we transformed gene expression values to gene set enrichment scores (ES) by mapping the genes on preselected groups of genes. Gene sets are groups of genes that have been experimentally and/or computationally related to a biological function. We applied this transformation in three unrelated cases (datasets) and demonstrated that this transformation can compensate for the technological differences between the microarray and RNA-Seq transcriptomics platforms resulting in a dramatic increase in concordance between them. Because of this increased concordance, we were able to show that models based on microarray platform data can be used on RNA-seq transcriptomic platforms data enabling meta-analysis over multiple studies. The transformation to gene sets is by definition a simplification of the biological complexity but is the price to pay for homogenizing in comparable data. The ability to create cross validated models and predict different subtypes of cancer after ES transformation with results equal or better than in earlier reported studies leads us to conclude that the relevant biological content is retained after this transformation.

Additionally, the transformation based on gene sets is meant to place single genes in a biological context. Therefore, we aimed to test whether our results were driven by the biological relationships captured in the gene sets. To do so, we performed permutations of the gene sets to purposely remove the biological information that is gathered in these sets. The results of the permutation test showed a significant decrease in the concordance between the RNA-Seq and microarray platform with respect to the results attained with non-permuted gene sets.

Part of the enrichment score transformation involves the conversion from gene expression levels to ranks. We tested whether the observed increase in concordance between platforms after gene set transformation was a result of this ranking alone. We found that a simple ranking of the raw expression values removed a nonlinear trend but the larger part of the gene expression remained non correlated between microarrays and RNA-Seq. The increased platform similarity therefore cannot be attributed to the ranking effect alone but is a combined effect of the biological information gathered in the gene sets and the rank-based gene set score.

Gene sets can be considered as *a priori* defined filters as gene expression levels are no longer considered individually but as a set. The number of genes within an individual gene set affects the stability and noise filtering capability of that gene set at the expense of impact for the individual genes in the set. For this reason, the number of and which gene sets to use in an analysis is a trade-off between resolution and noise. We feel further research is needed to elucidate the role of individual gene set sizes and the number of necessary sets to obtain the data transformation while maintaining enough resolution and noise filtering capability. Liberzon et al. [18] have highlighted the intrinsic redundancy and heterogeneity with the large number of gene-sets. Many high-scoring but overlapping gene-sets can hide potentially relevant hits further down the list. More cumbersome however, are genes in a given set that do not behave consistently, effectively rendering the gene-set non-informative. Using expert human biological review proved essential for curating and selecting the right ‘hallmark’ gene-set.

To conclude, it is considered that RNA-Seq will fully replace the microarray platform as RNA-Seq costs continue to drop. However, the amount of microarray data that is already available should not be disregarded. We showed in this study that although the microarray and RNA-Seq transcriptomics platforms can show conflicting results at gene level, there is a high degree of concordance between the platforms after gene set enrichment score transformation. We conclude that this transformation therefore enables data integration of the two platforms. The mixed platform data results in larger datasets and may aid studies in obtaining statistical significance. This way, the mRNA data of valuable and often unique experiments can be re-used.

## Supporting information

S1 Fig Schematic overview of samples in dataset 1. (TIF)

S2 Fig Schematic overview of samples in dataset 2. (TIF)

S3 Fig Schematic overview of samples in dataset 3. (TIF)

S4 Fig Schematic overview of the groups (ES_*MA*_ and ES_*SEQ*_) in dataset 3 used for modelling. (TIF)

S1 Text Enrichment scores of data with many zeros. (DOCX)
