# Supplementary figures and images for: Increased comparability between RNA-Seq and microarray data by utilization of gene sets
Source: PLoS Comput Biol. 2020 Sep 30;16(9):e1008295. doi: 10.1371/journal.pcbi.1008295 (PMC7549825; doi:10.1371/journal.pcbi.1008295)

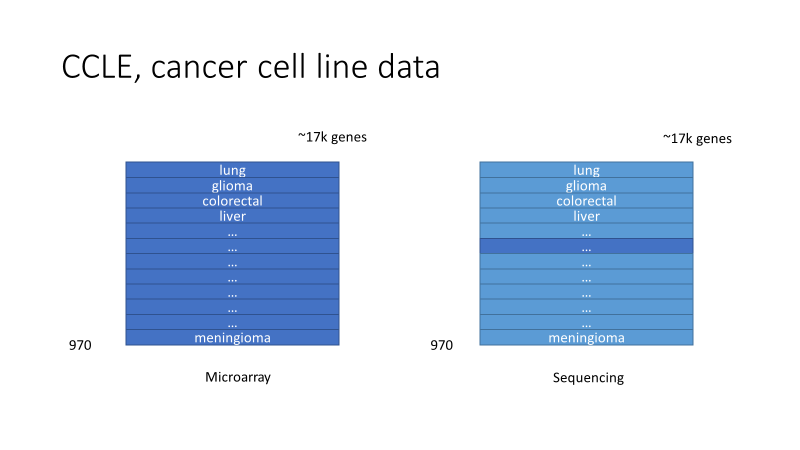

Supplement: S1 Fig — (TIF) [file pcbi.1008295.s001.tif]

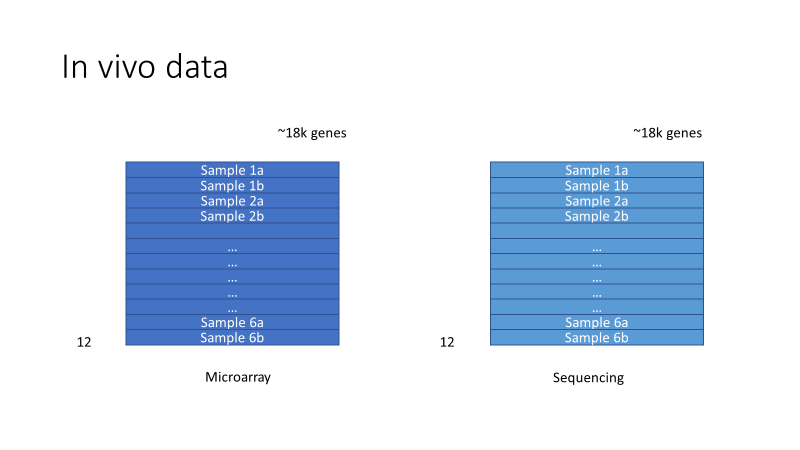

Supplement: S2 Fig — (TIF) [file pcbi.1008295.s002.tif]

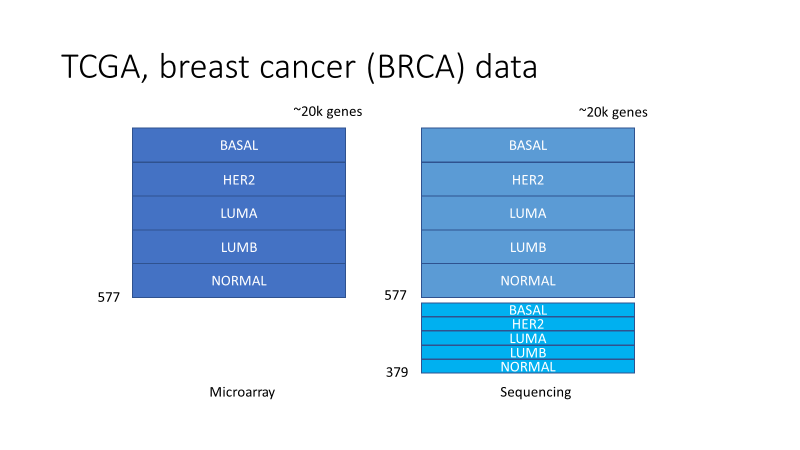

Supplement: S3 Fig — (TIF) [file pcbi.1008295.s003.tif]

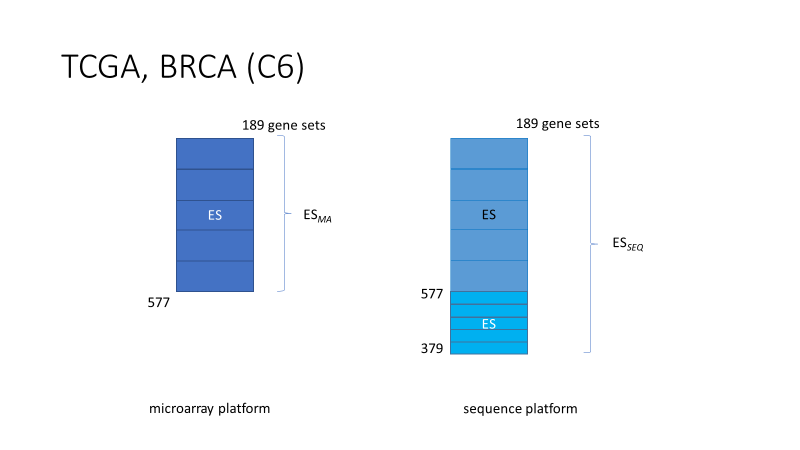

Supplement: S4 Fig — (TIF) [file pcbi.1008295.s004.tif]
