# Supplementary material for: Increased comparability between RNA-Seq and microarray data by utilization of gene sets
Source: PLoS Comput Biol. 2020 Sep 30;16(9):e1008295. doi: 10.1371/journal.pcbi.1008295 (PMC7549825; doi:10.1371/journal.pcbi.1008295)
Supplement: S1 Text — (DOCX) [file pcbi.1008295.s005.docx]

Supplementary to Increased comparability between RNA-Seq and microarray data by utilization of gene sets

*Frans M. van der Kloet^1^, Jeroen Buurmans^1^, Martijs J. Jonker^1^, Age K. Smilde^1^ and Johan A. Westerhuis^1^*^,🖂^

^1^ Swammerdam Institute for Life Sciences, University of Amsterdam

^🖂^ E-mail: j.a.westerhuis@uva.nl

# Enrichment scores of data with many zeros

The microarray based enrichment scores (ES*_MA_*) compared to sequence based enrichment scores (ES*_SEQ_*) show an offset (difference). Where the lowest value for ES*_SEQ_* is approximately -500, the value for the same sample in ES*_MA_* is -3000 (see Figure S5 1).

| 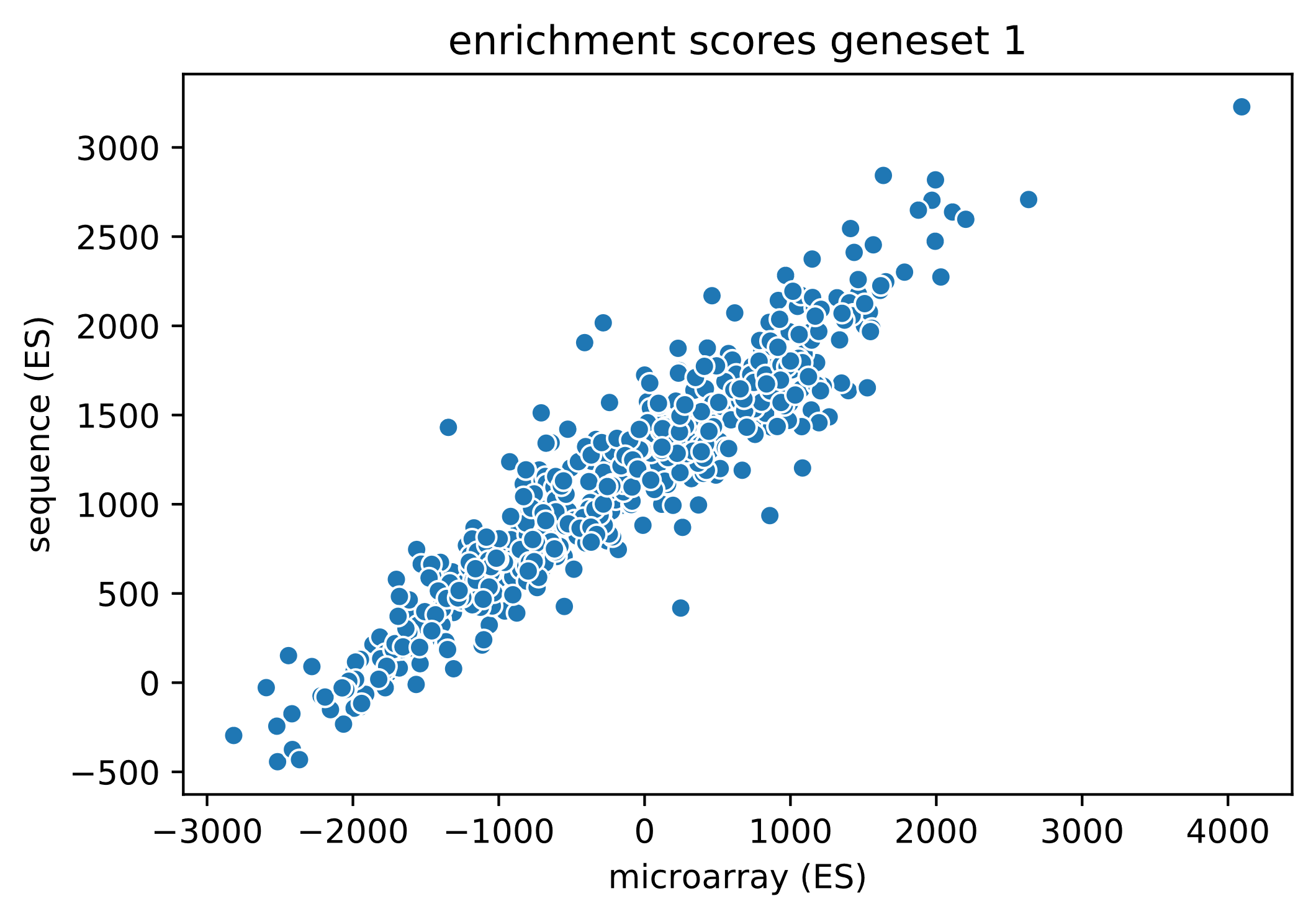 |
| --- |
| Figure S5 1. The offset between enrichment scores based on microarray data from data set 2 and enrichment scores based on sequence data from that same data set. |

This difference can partially be contributed to, of course, the rank difference between the genes. In Figure S5 2, the order of the genes for an arbitrary sample (TCGA-A8-A06Q-01) in the sequencing data were reordered according to the observed rank ordering of the microarray data for the same sample. In a perfect case, the scores would be exactly the same. Even though the values of the scores are of the same order, the offset is still observed (e.g. -5200 vs -4300). Notably, the sequence based scores showed a positive offset with respect to the microarray based scores.

| 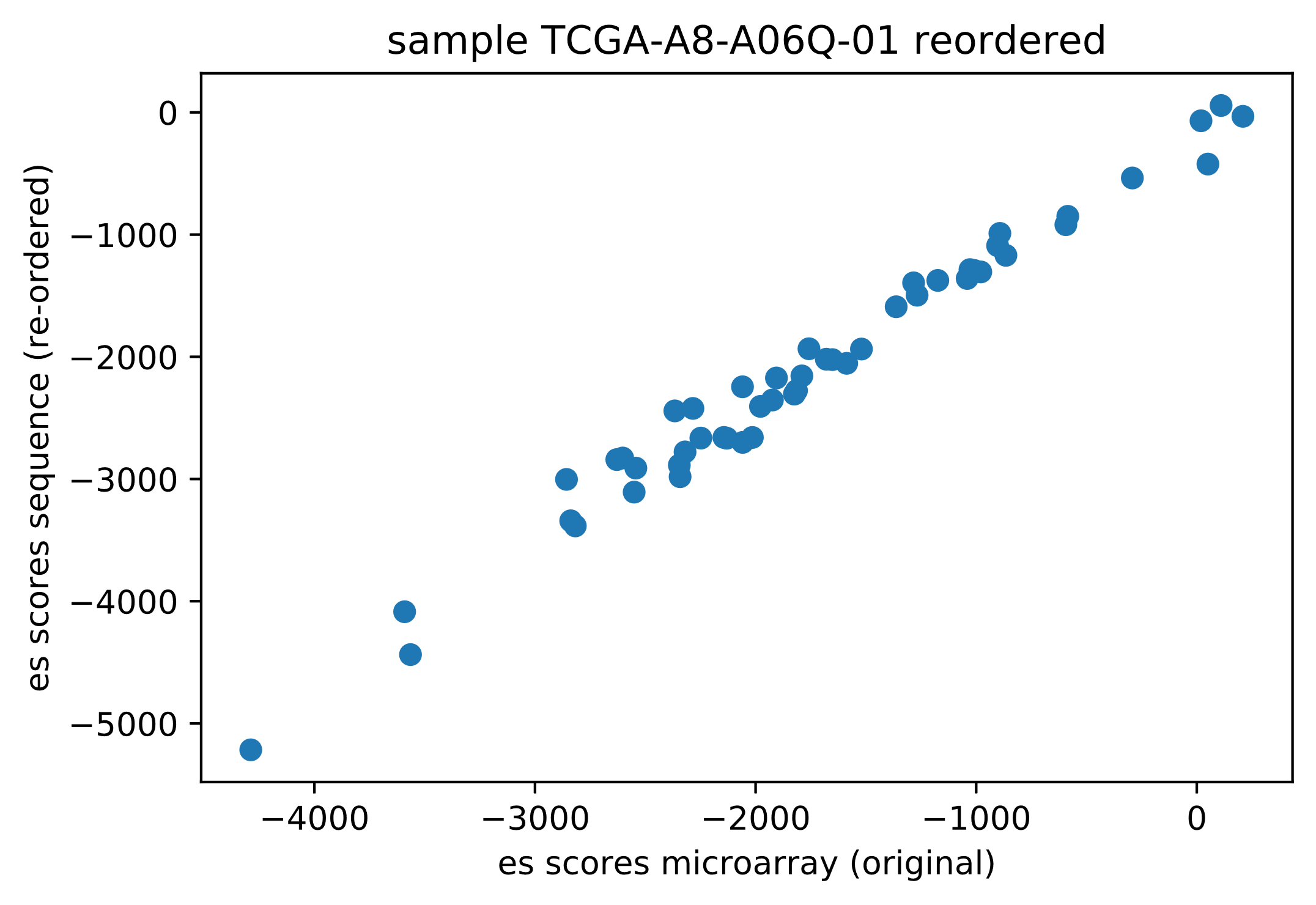 |
| --- |
| Figure S5 2. Enrichment scores (H-collection) for the same sample after reordering the gene ranks of the sequence data to correspond to that of the microarray data. |

Figures S5 3 A and B show the observed rank distribution for all genes of that same arbitrary sample for the microarray and sequence data respectively. Genes that have the same rank have an average rank value.

| 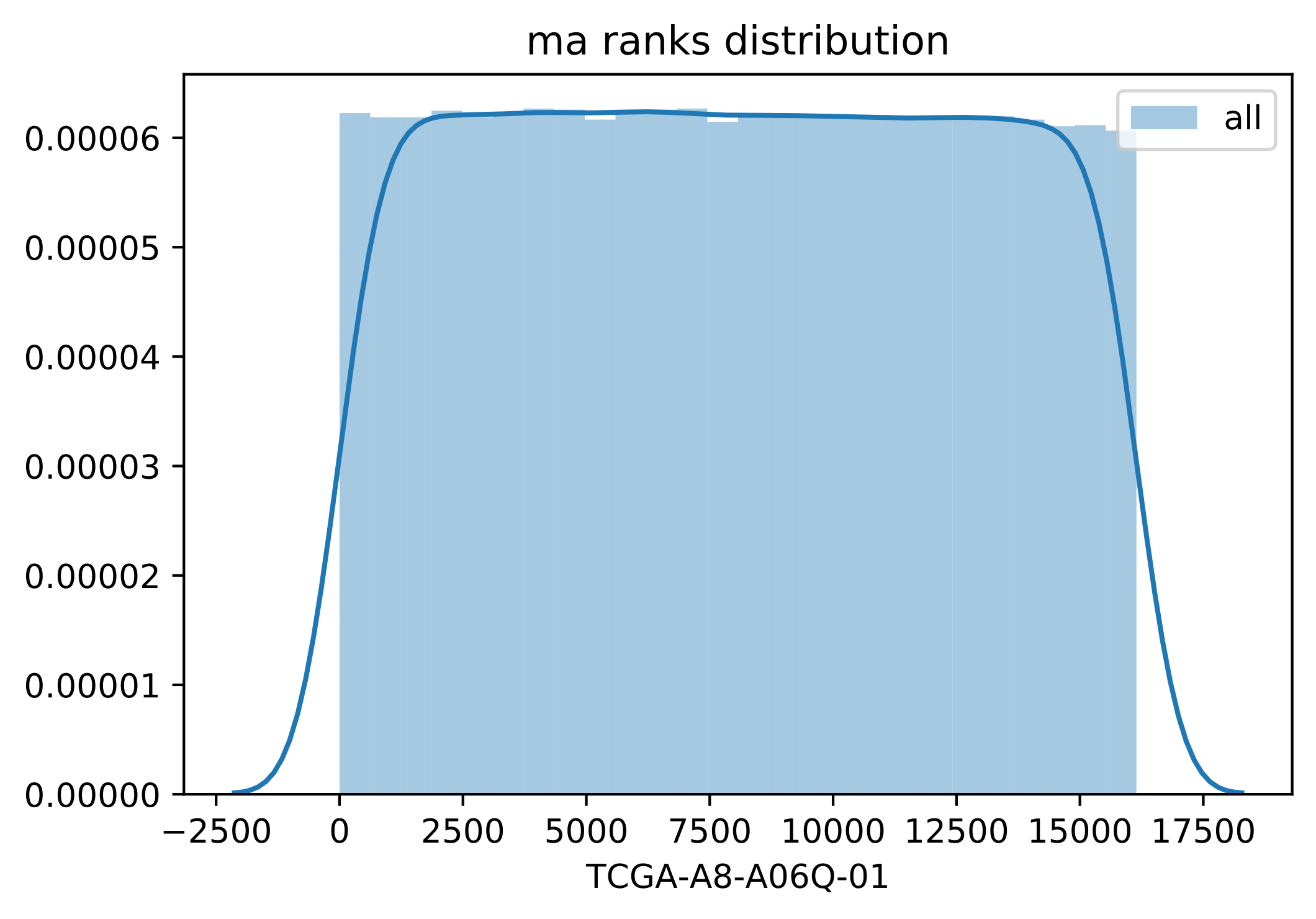 | 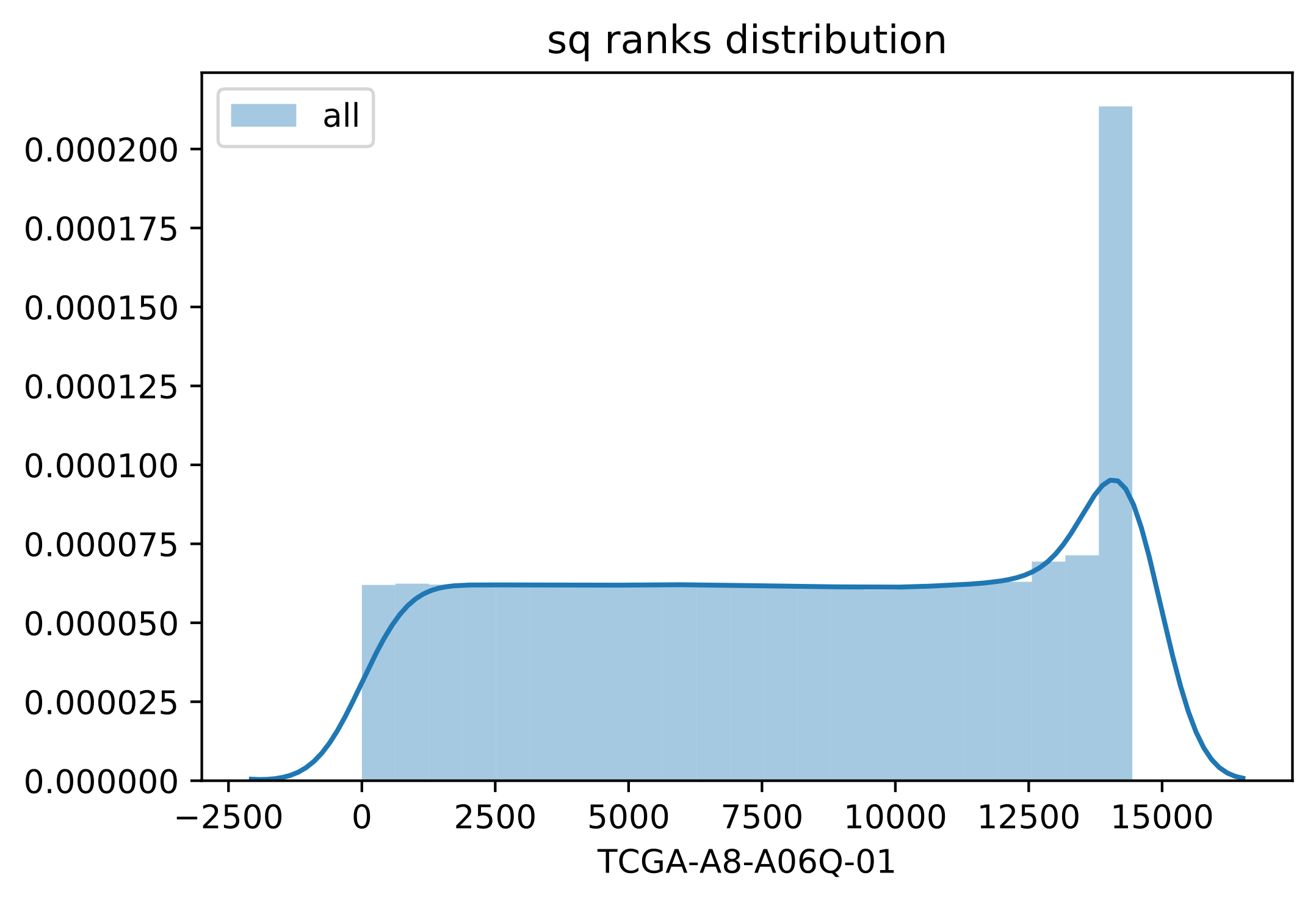 |
| --- | --- |
| Figure S5 3. A, the distribution of the ranks found in a typical (arbitrary) microarray sample in data set 2. B, the distribution of the ranks found in the sequence data for the same sample as in A. The blue lines indicate the fitted density function. | |

The ranks of the microarray data (on the left) shows a clear uniform distribution. The sequencing data on the right however, shows a large number of low ranked genes (i.e. genes with the same number of low counts, in our case ones). Because the enrichment score (ES) is a rank based score this most likely will be affected by a large number of equally ranked values. To investigate this effect we artificially added many low ranked genes (zeros) to our data. None of these added genes were part of any gene set. Adding these genes does not change the rank of the original genes but does affect the separate parts ($P_{H}$ and $P_{NH}$) of the enrichments score function. The overall effect is clearly visible In Figure S5 4 A; the enrichment scores for the H-collection (for a single sample) are elevated albeit not uniform. The scores increase with increasing levels of added zeros. When artificially increasing the data by adding high ranked genes (i.e. maximum values) the enrichment scores are affected uniformly but lowered.

| 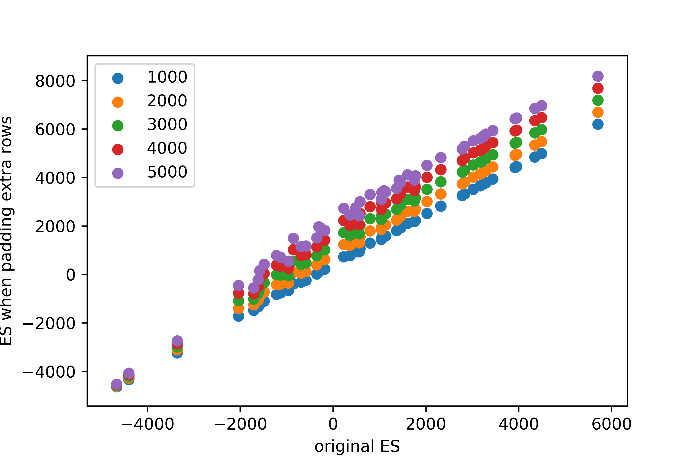 | 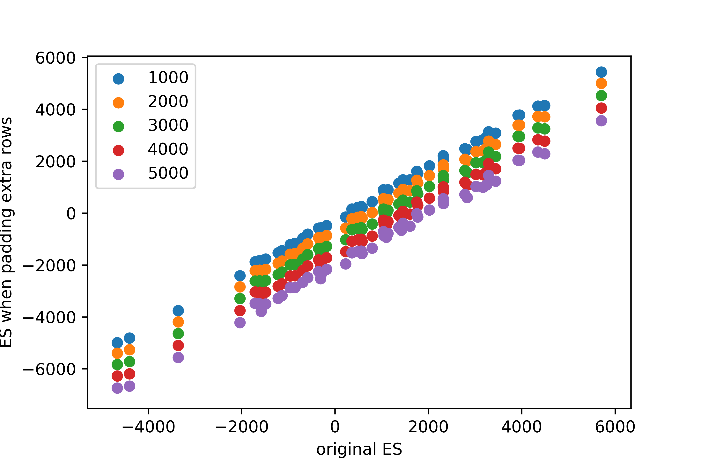 |
| --- | --- |
| Figure S5 4. A, The non-uniform effect of artificially adding low ranked genes to the data for the enrichment scores (H-collection, 50 scores) of a sample. B, The uniform lowering effect of artificially adding high ranked genes to the data on the enrichment scores. | |

The additive effect of adding lower ranked values finds its origin in the lowered values of $P_{NH}$vector while the values in $P_{H}$ are invariant to adding low ranked genes. In Figure S5 A the values in the $P_{H}$ vector for the genes in a single gene set (from collection h) are plotted for a single sample. Figure S5 5B shows the corresponding values in the $P_{NH}$ vector for the same sample. The enrichment score is the sum $\sum_{r=1}^{R} \left( P_{H}-P_{NH} \right)$over all the genes and consequently increases with increasing number of low ranked values.

| 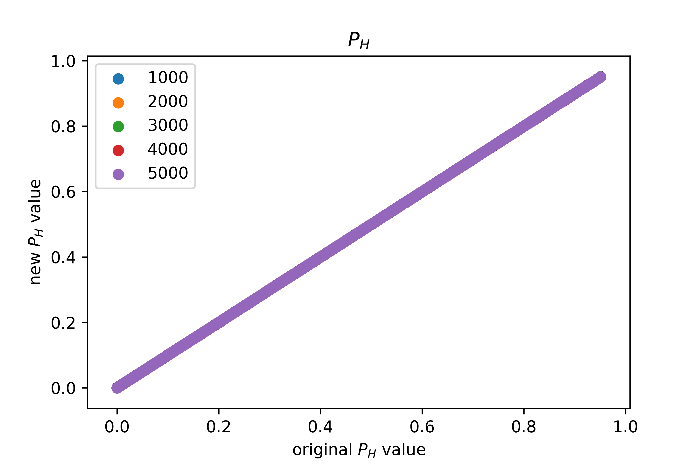 | 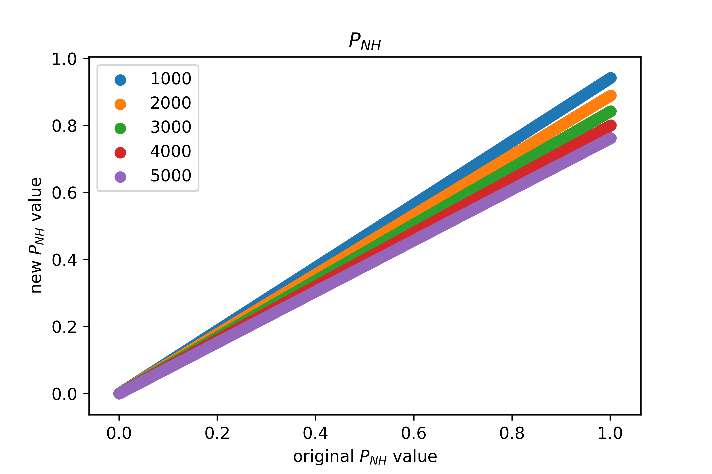 |
| --- | --- |
| Figure S5 5. A, The $P_{H}$vector is invariant to adding different numbers of low ranked genes. B, The corresponding values in the $P_{NH}$ vector are lower with increasing number of low ranked genes. | |

The negative effect of adding higher ranked values has a combined origin. In this case the $P_{H}$values are shifted to the right (see Figure S5 6A) and the $P_{NH}$ values (Figure S5 6B) are lowered with increasing number of added high ranked values. The effect of the horizontal shift is so large that the overall sum of the difference between $P_{H}$and $P_{NH}$for all genes, the enrichment score, is lower with increasing number of high ranked values.

| 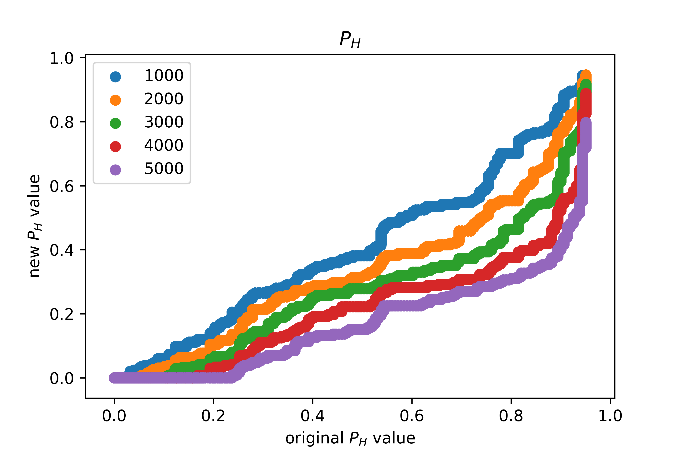 | 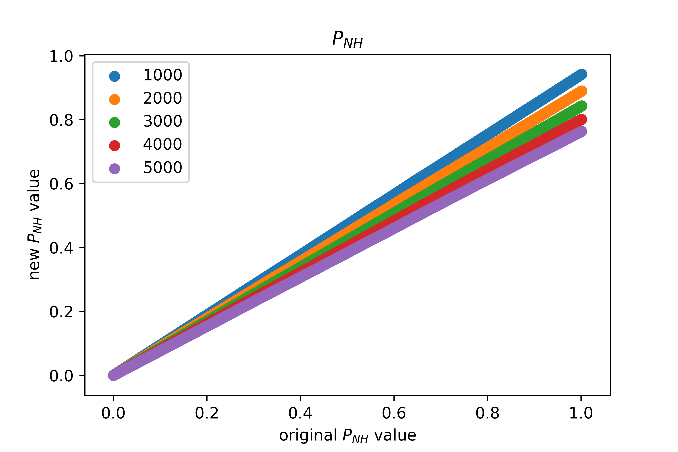 |
| --- | --- |
| Figure S5 6. A, The $P_{H}$vector shifts to the right with increasing number of added high ranked values. B, The corresponding values in the $P_{NH}$vector are lower with increasing number of high ranked genes. | |

Because of the abundant number of low ranked genes ( those with low value) in the sequence data of data set 2 (Figure S5 3B) we argue that this is the main cause of the observed offset difference in Figure S5 1 which introduces a non-uniform kind of offset (Figure S5 4A). Commonly, sequence data will contain many genes with low read counts. One way to deal with this would be to use a cut-off filter and remove those genes with these low counts.

In general it can be concluded that the enrichment score is sensitive to the number of values with same ranks.
